# Supplementary figures and images for: Ultrastructural Study and Immunohistochemical Characteristics of Mesencephalic Tegmentum in Juvenile Chum Salmon (Oncorhynchus keta) Brain After Acute Traumatic Injury
Source: Int J Mol Sci. 2025 Jan 14;26(2):644. doi: 10.3390/ijms26020644 (PMC11765592; doi:10.3390/ijms26020644)

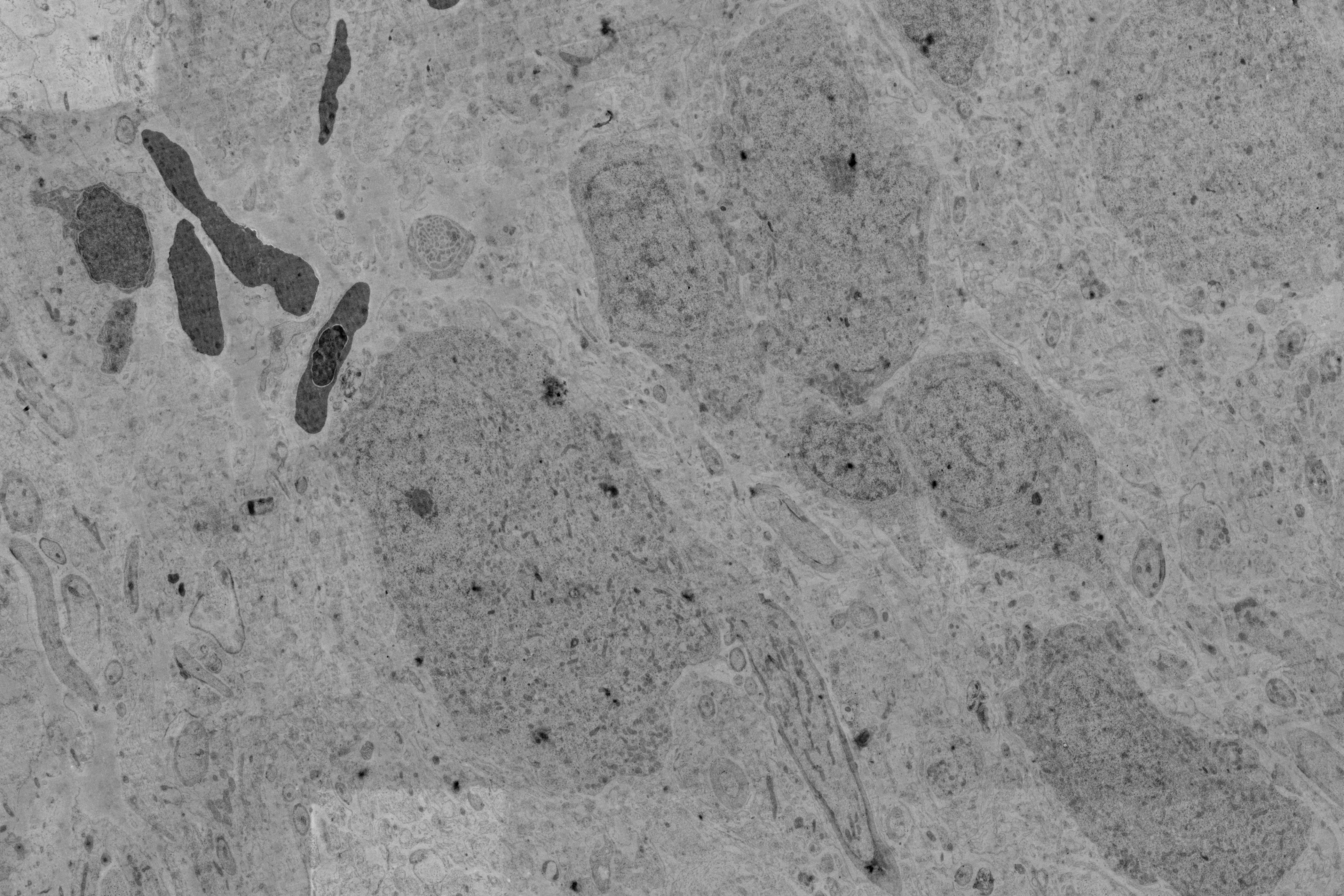

Supplement: Supplementary file 1 [file ijms-26-00644-s001.zip › Figures TEM without color musk/Figure 2/2A.tif]

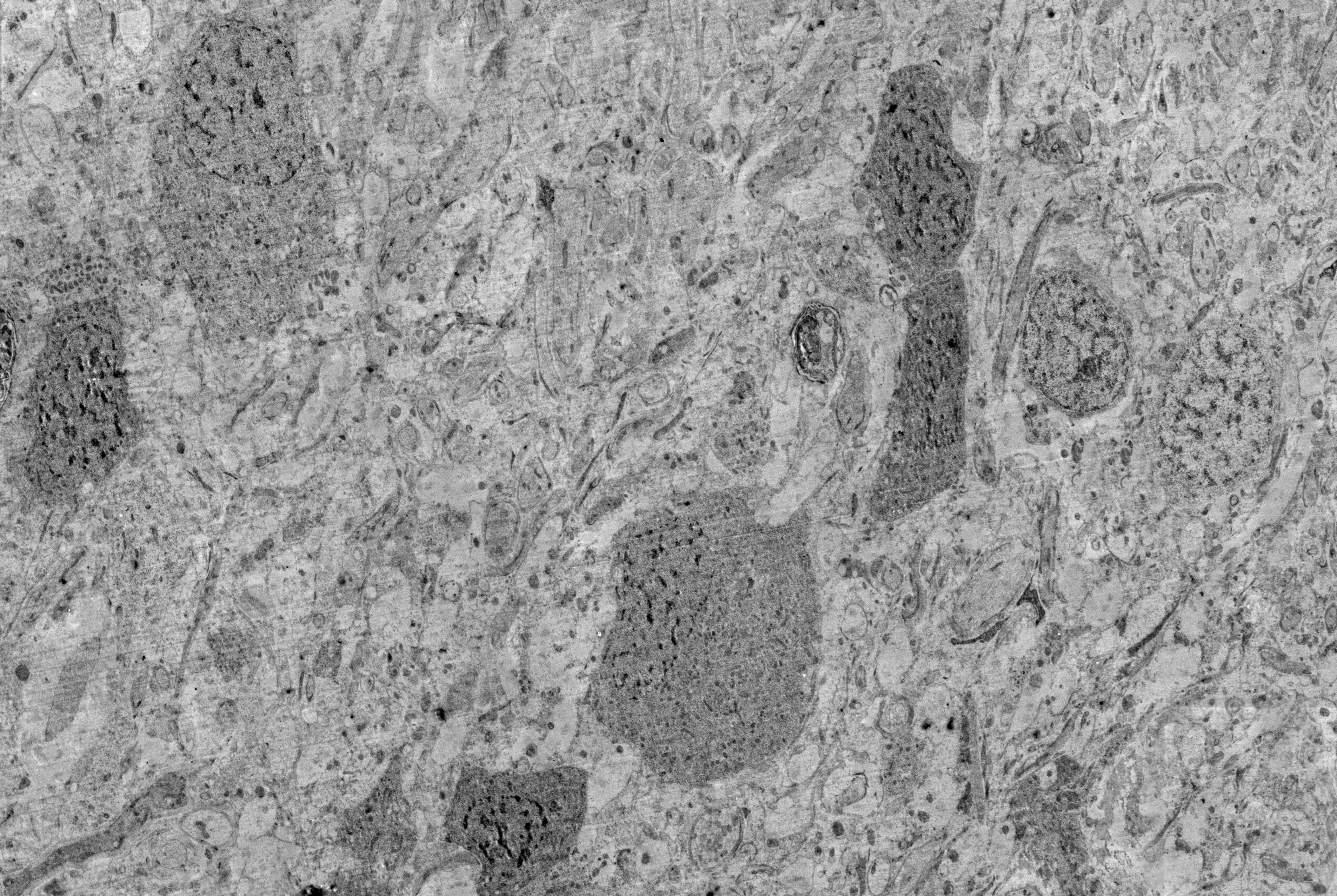

Supplement: Supplementary file 1 [file ijms-26-00644-s001.zip › Figures TEM without color musk/Figure 3/3A.tif]

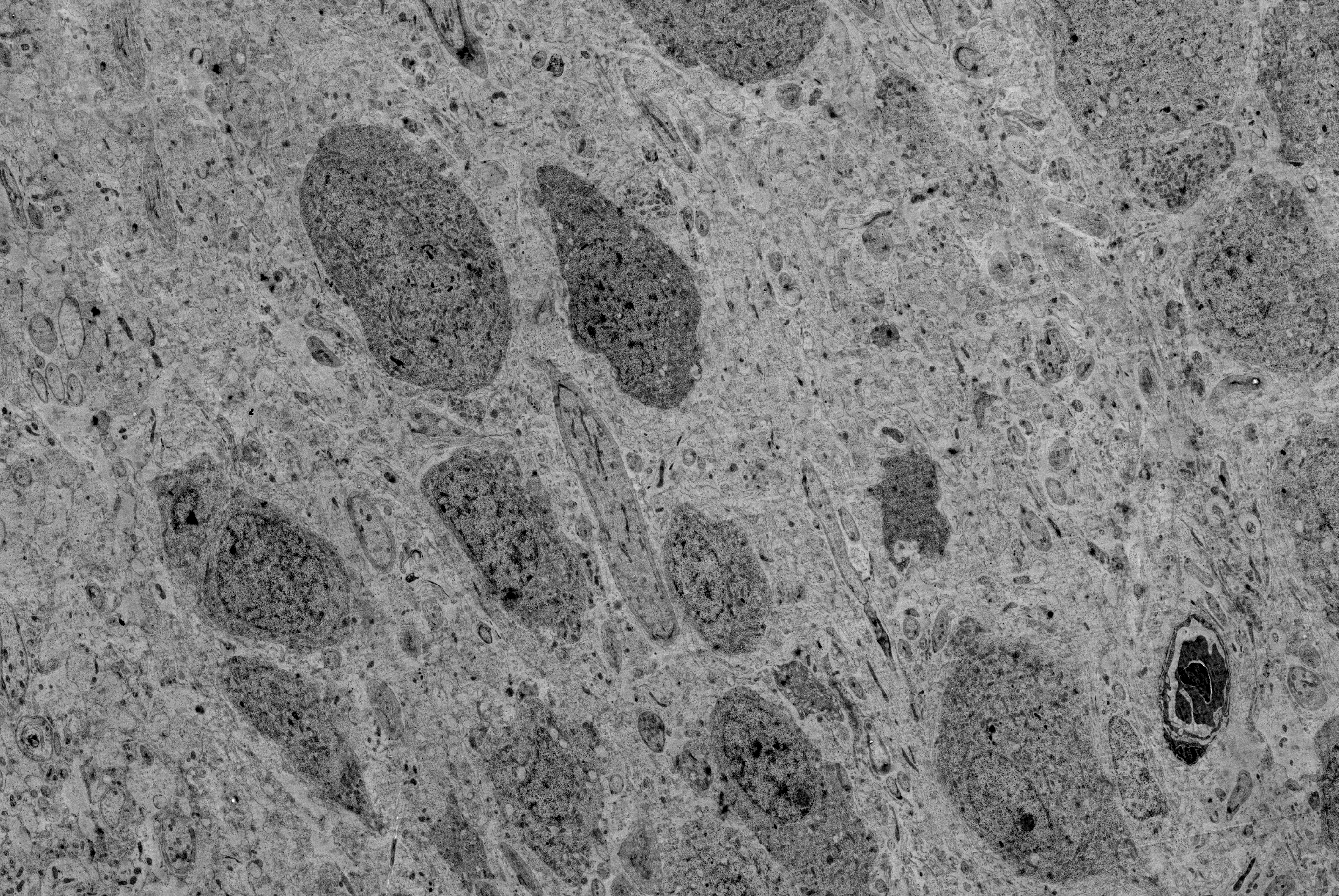

Supplement: Supplementary file 1 [file ijms-26-00644-s001.zip › Figures TEM without color musk/Figure 3/3B.tif]

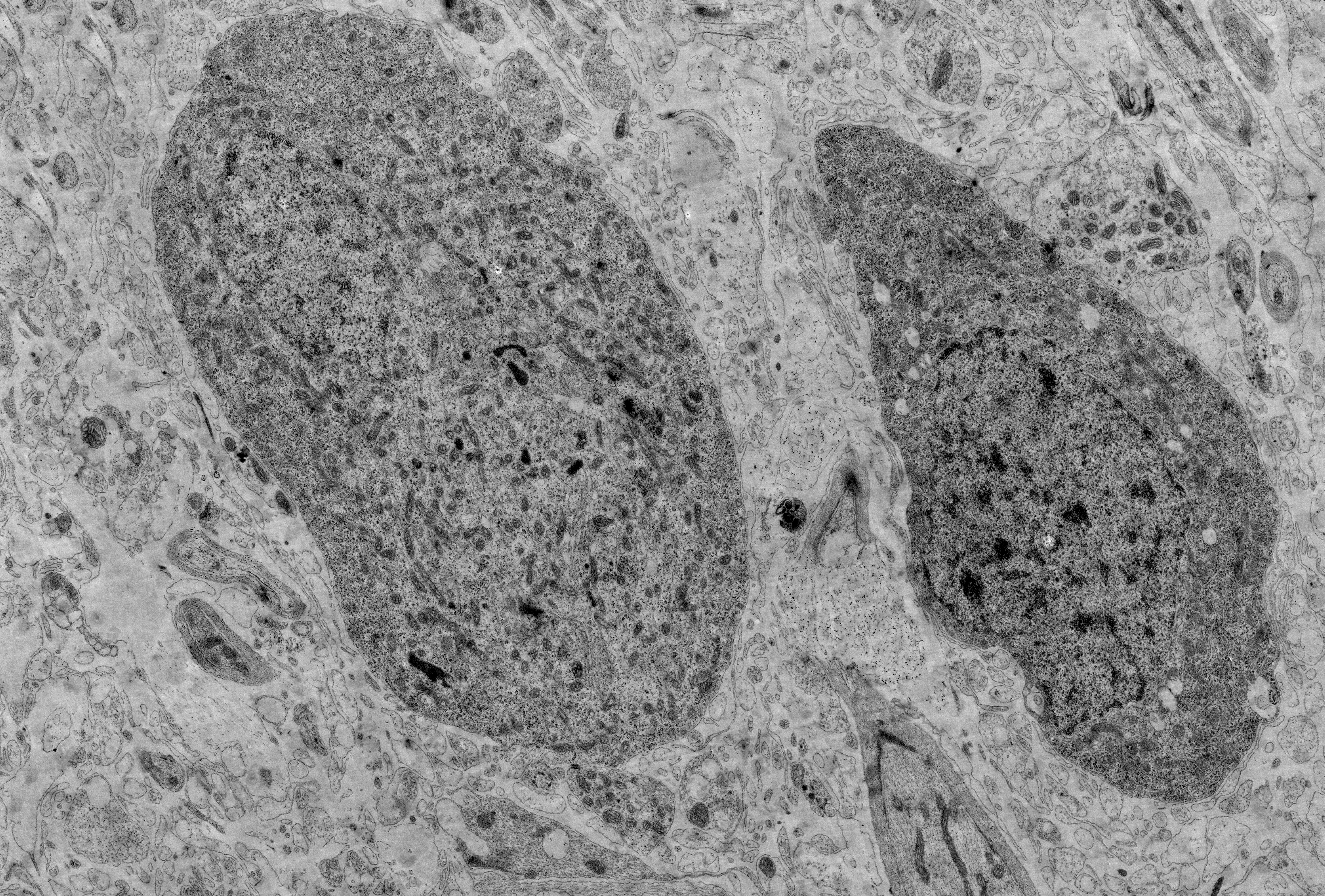

Supplement: Supplementary file 1 [file ijms-26-00644-s001.zip › Figures TEM without color musk/Figure 3/3C.tif]

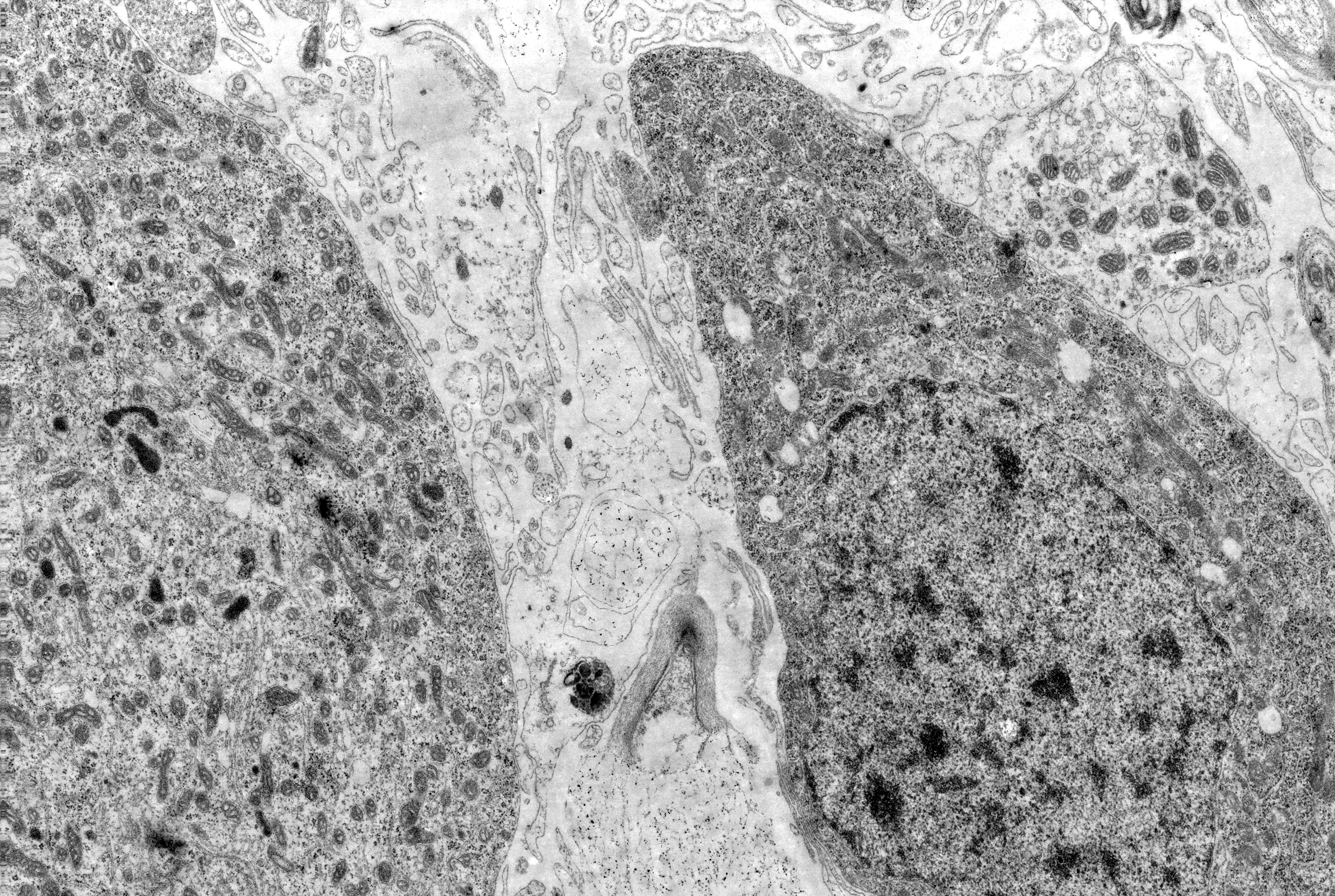

Supplement: Supplementary file 1 [file ijms-26-00644-s001.zip › Figures TEM without color musk/Figure 3/3D.tif]

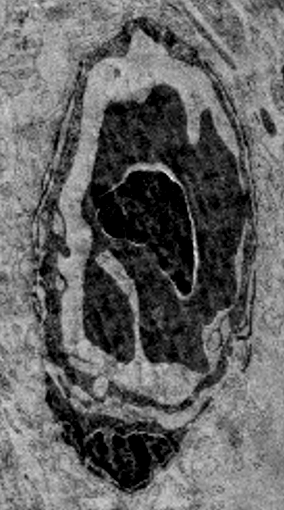

Supplement: Supplementary file 1 [file ijms-26-00644-s001.zip › Figures TEM without color musk/Figure 3/3B2.tif]

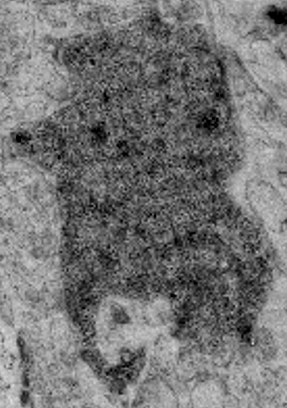

Supplement: Supplementary file 1 [file ijms-26-00644-s001.zip › Figures TEM without color musk/Figure 3/3B1.tif]

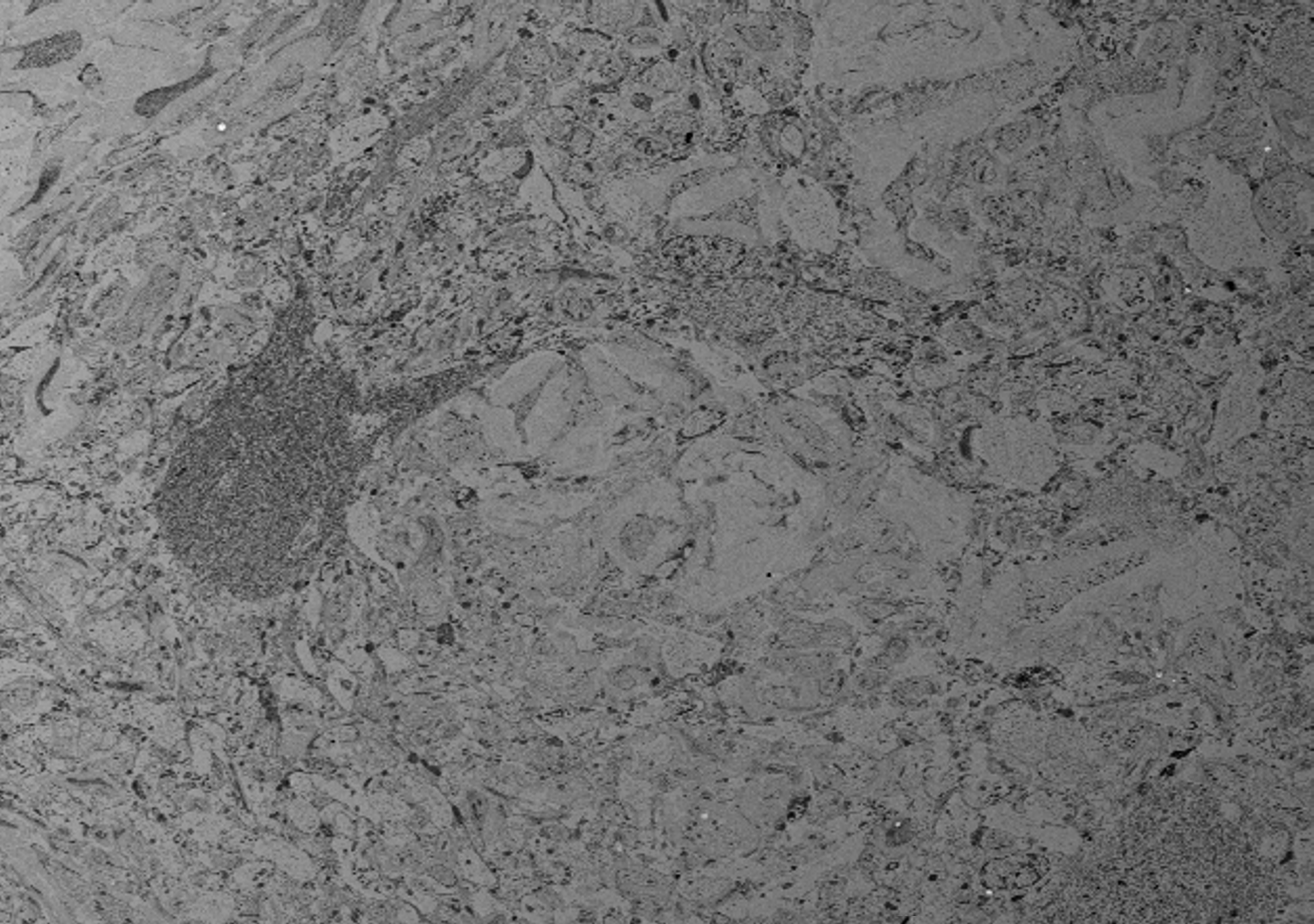

Supplement: Supplementary file 1 [file ijms-26-00644-s001.zip › Figures TEM without color musk/Figure 4/4A.tif]

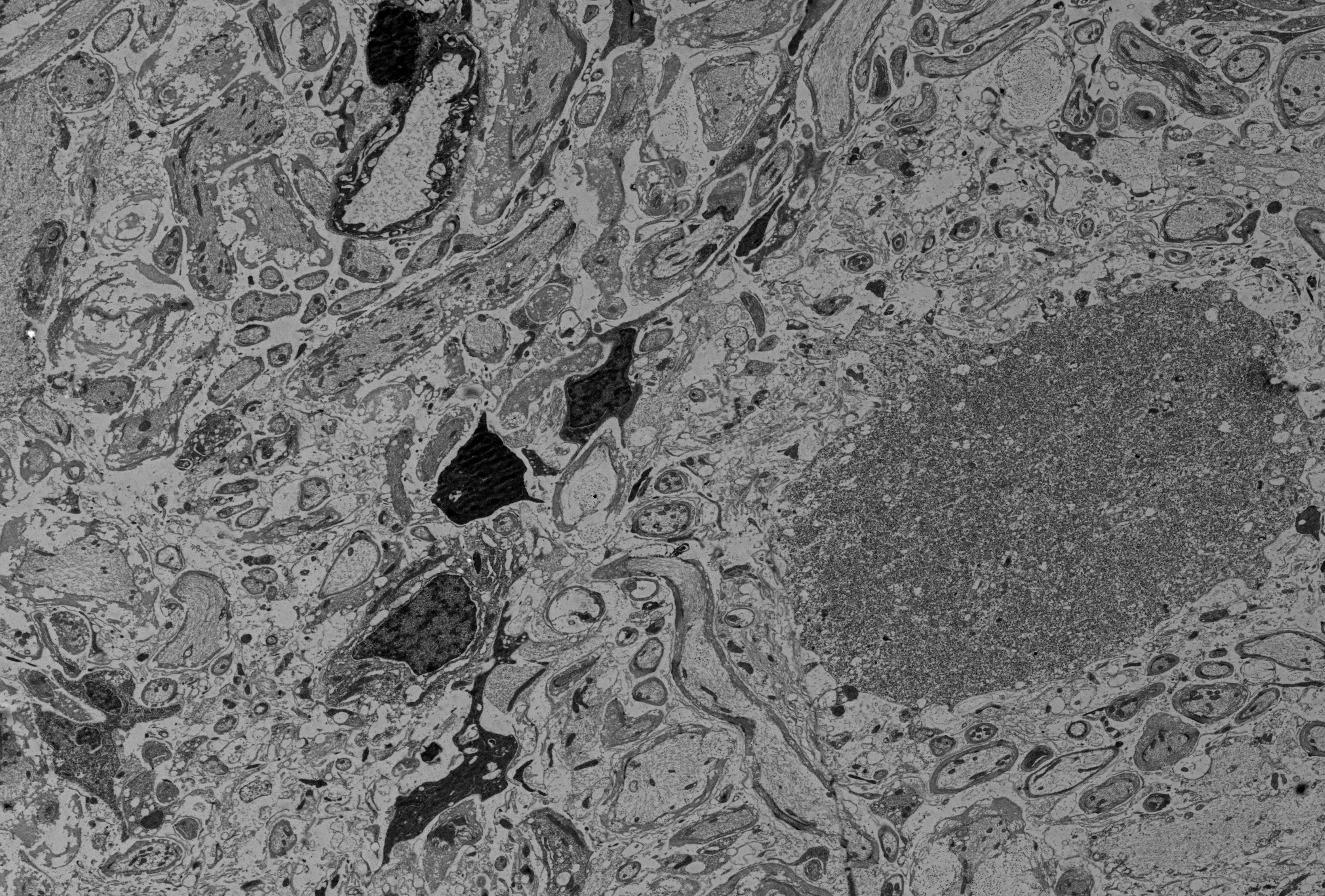

Supplement: Supplementary file 1 [file ijms-26-00644-s001.zip › Figures TEM without color musk/Figure 4/4B.tif]

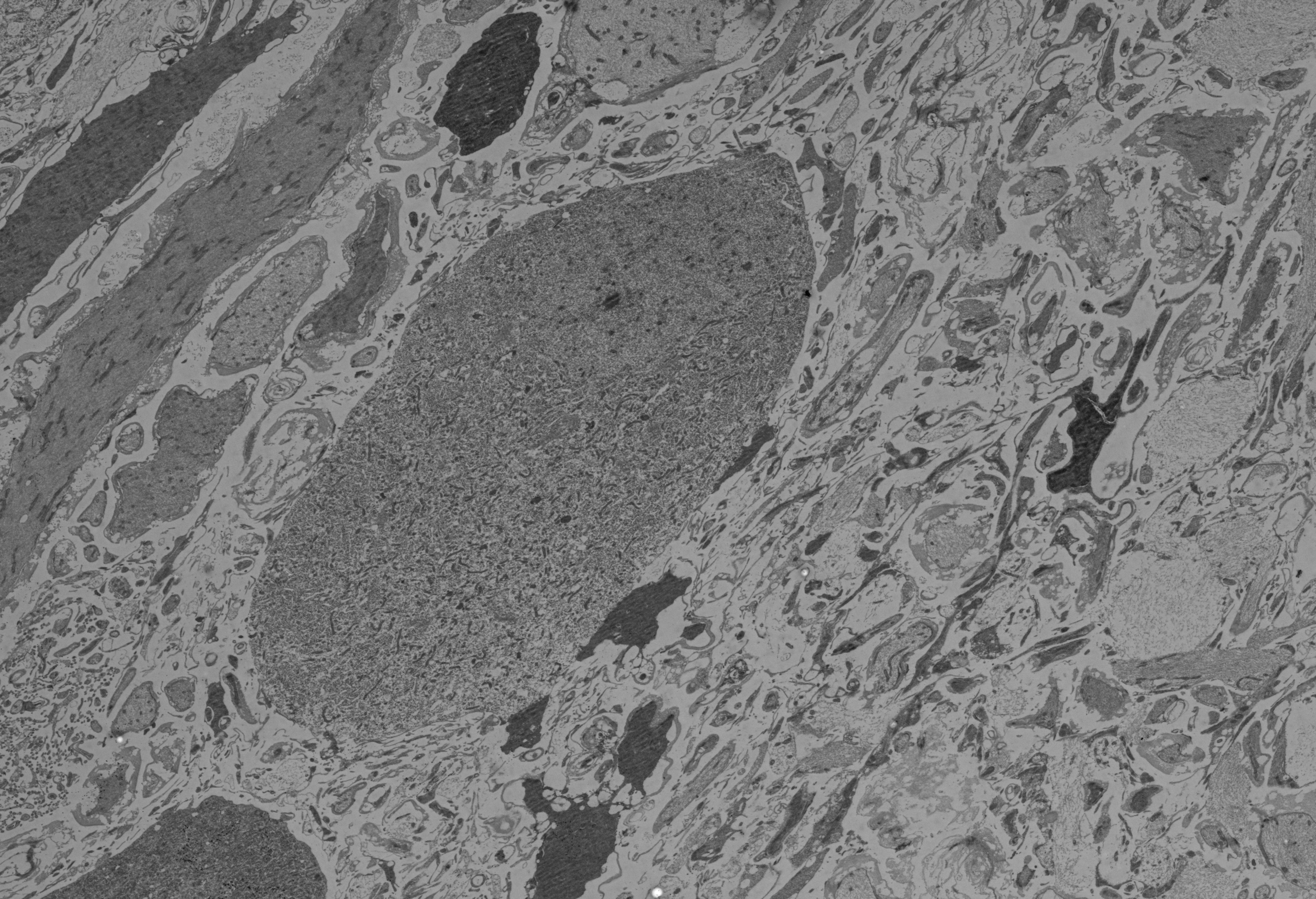

Supplement: Supplementary file 1 [file ijms-26-00644-s001.zip › Figures TEM without color musk/Figure 4/4C.tif]

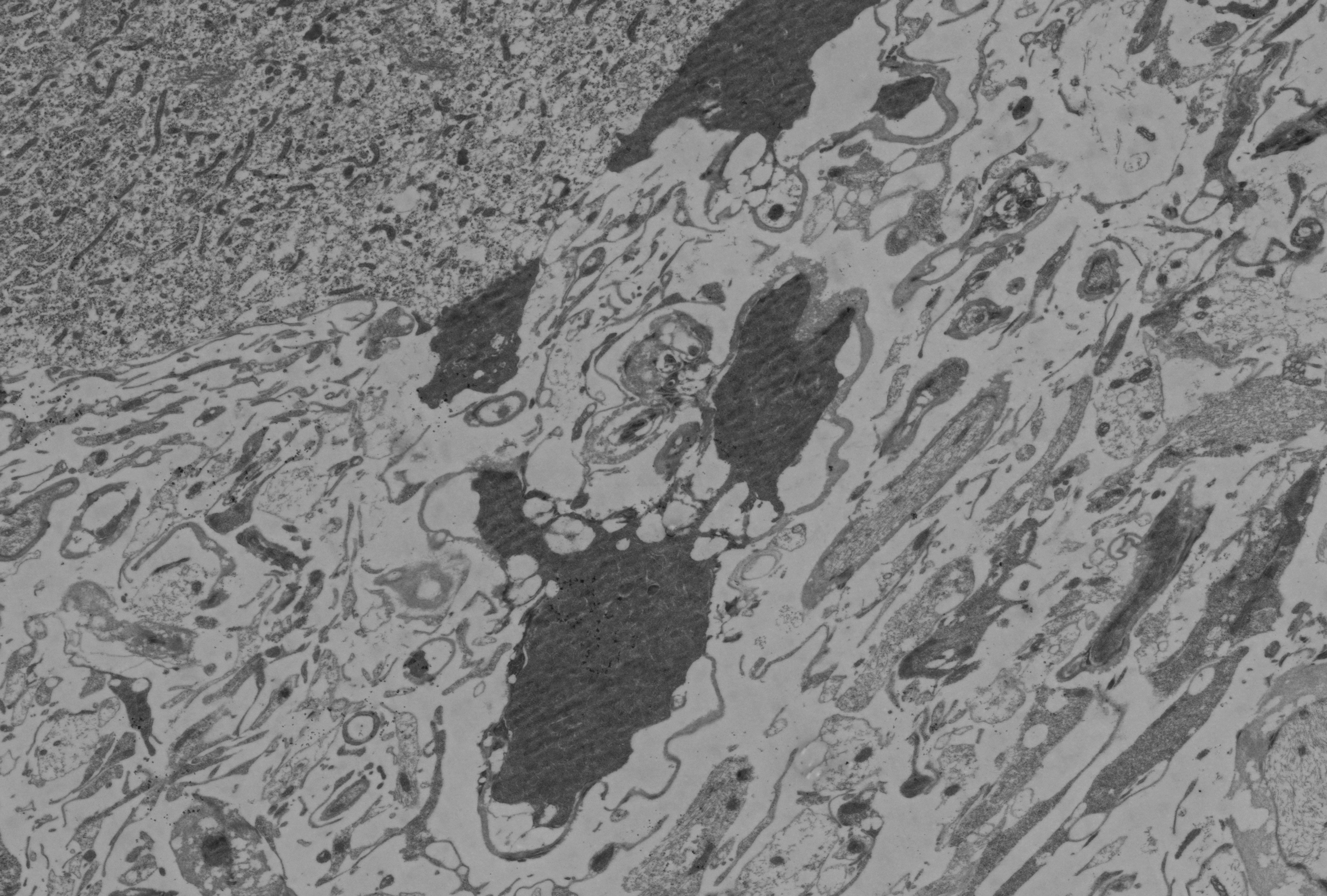

Supplement: Supplementary file 1 [file ijms-26-00644-s001.zip › Figures TEM without color musk/Figure 4/4D.tif]

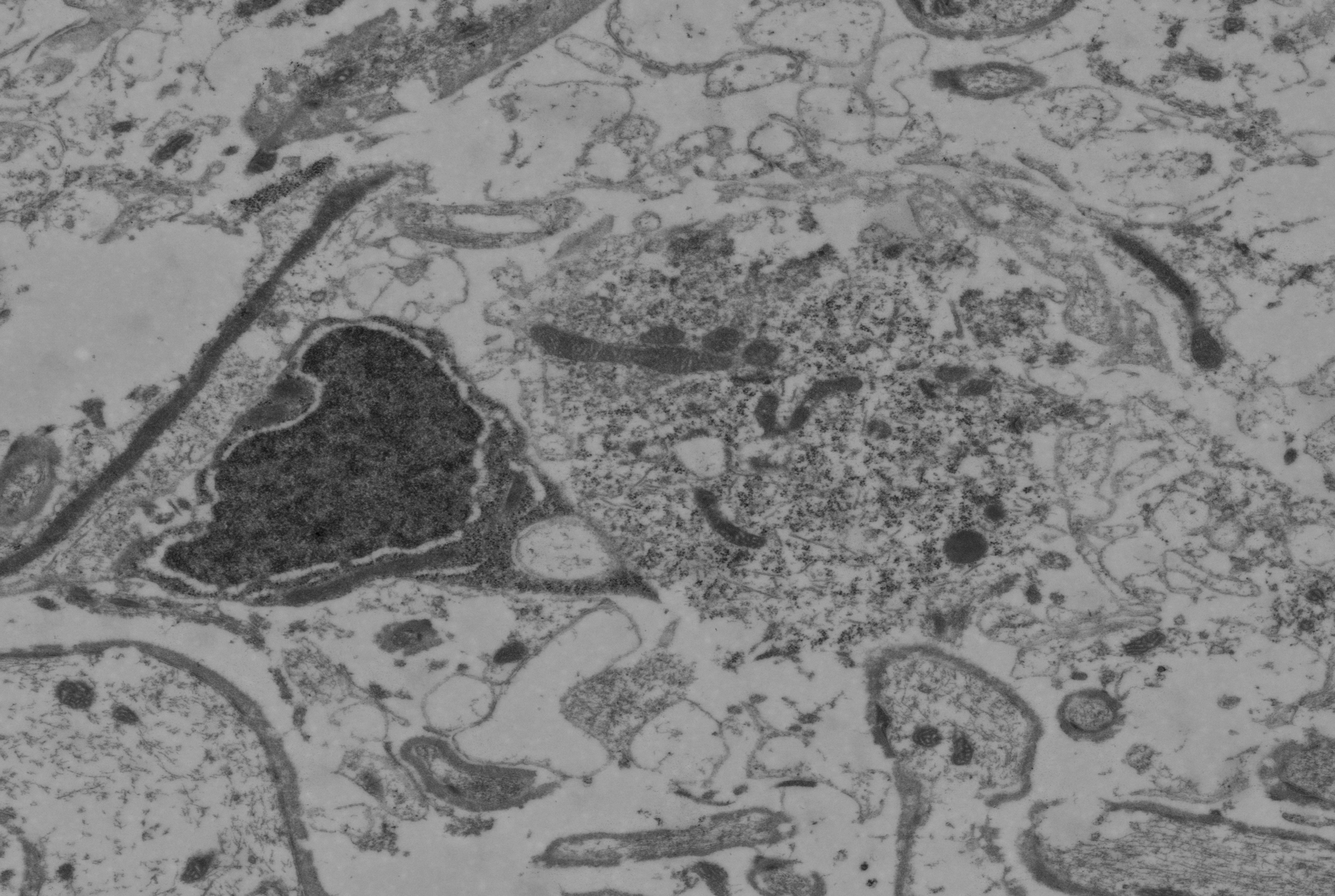

Supplement: Supplementary file 1 [file ijms-26-00644-s001.zip › Figures TEM without color musk/Figure 4/4F.tif]

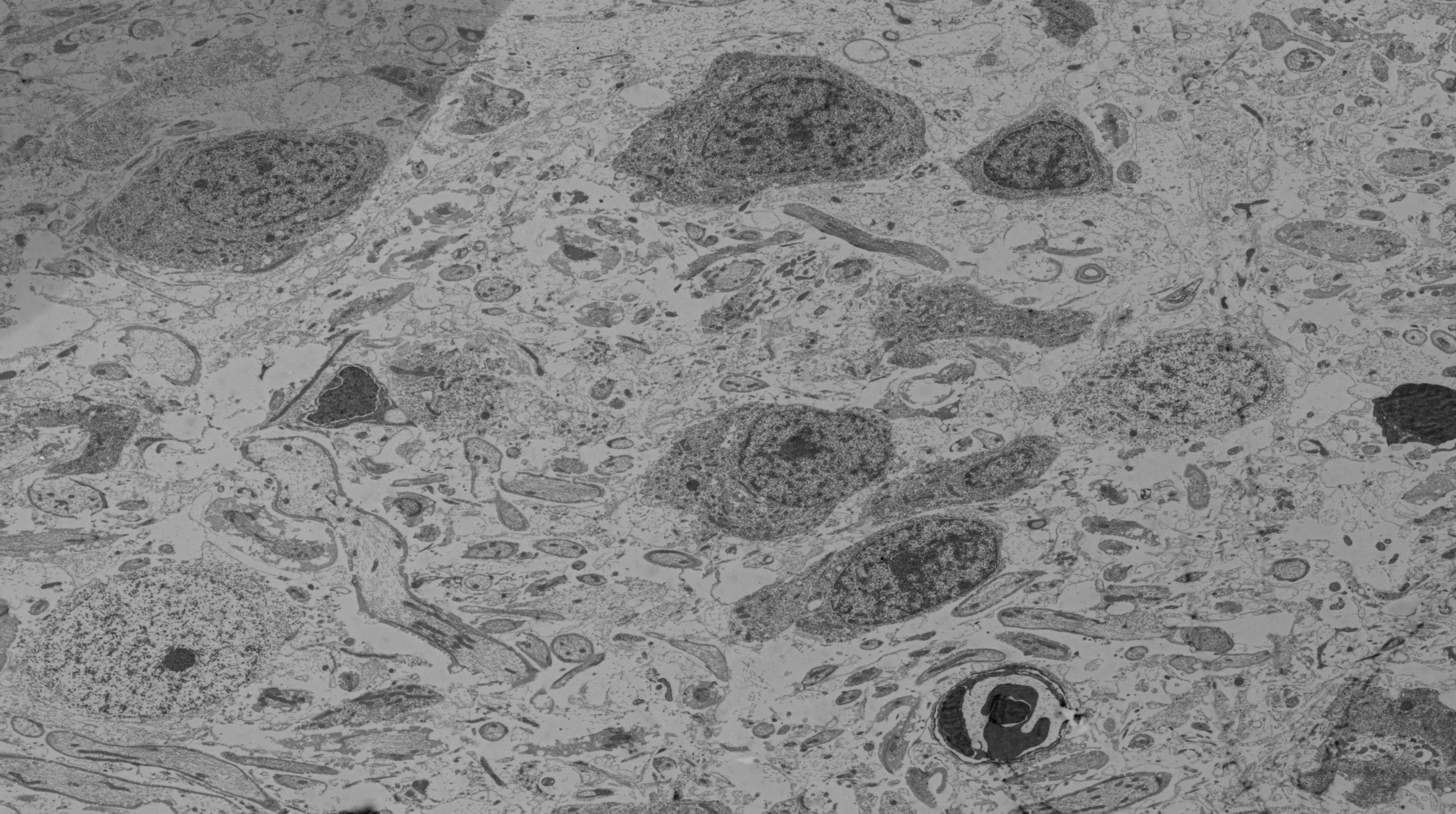

Supplement: Supplementary file 1 [file ijms-26-00644-s001.zip › Figures TEM without color musk/Figure 4/4G.tif]

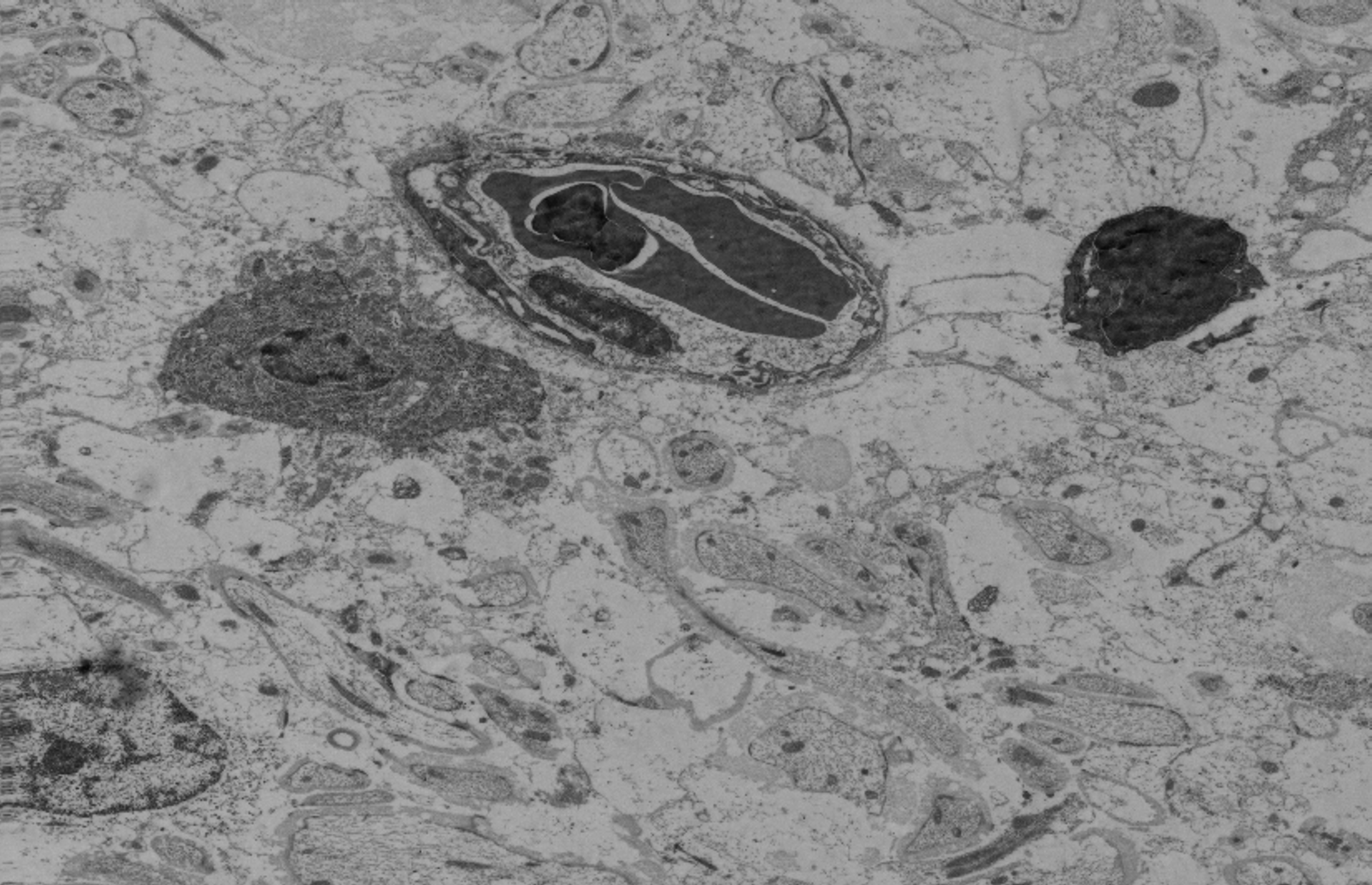

Supplement: Supplementary file 1 [file ijms-26-00644-s001.zip › Figures TEM without color musk/Figure 4/4H.tif]

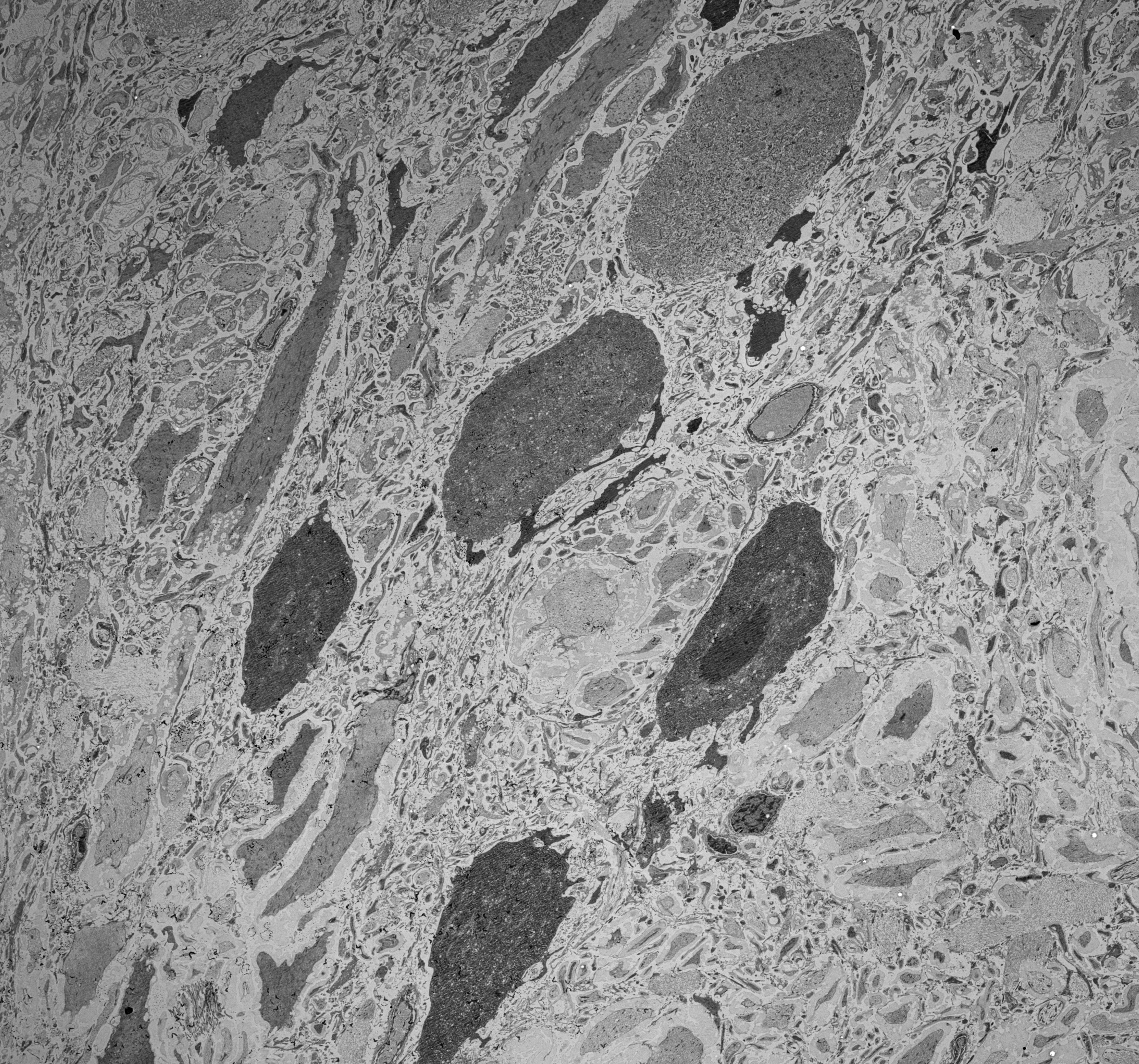

Supplement: Supplementary file 1 [file ijms-26-00644-s001.zip › Figures TEM without color musk/Figure 5/5A.tif]

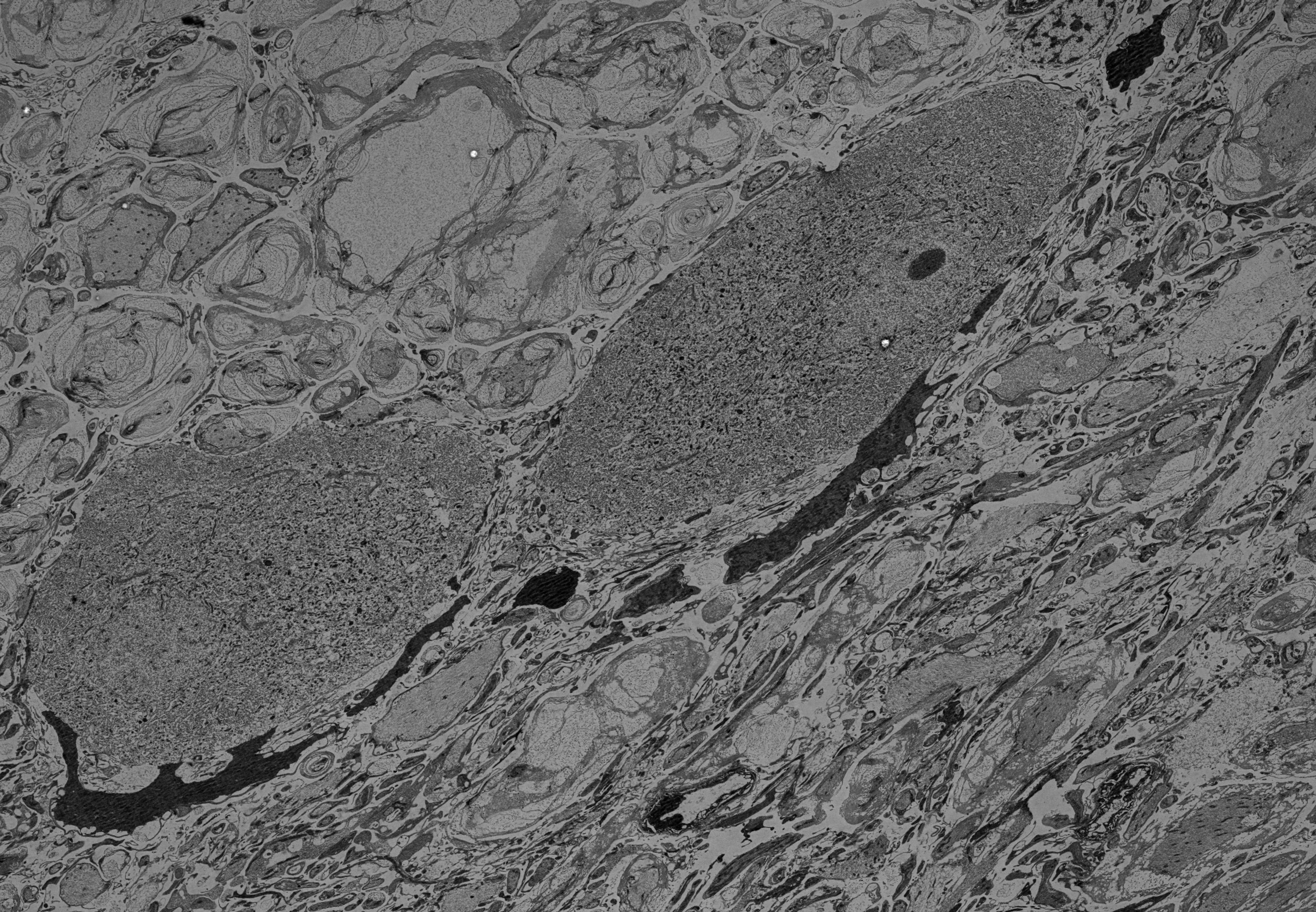

Supplement: Supplementary file 1 [file ijms-26-00644-s001.zip › Figures TEM without color musk/Figure 5/5E.tif]

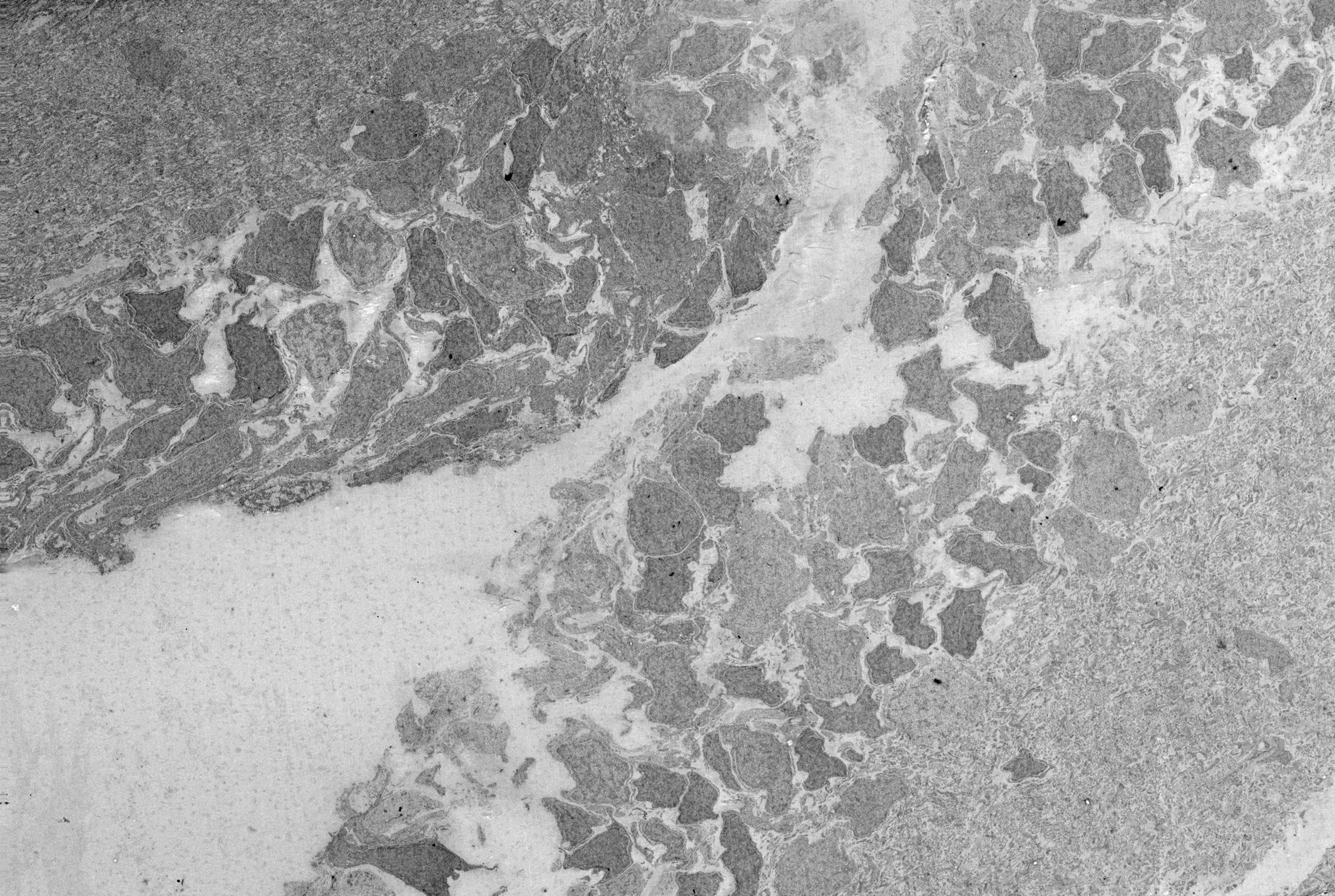

Supplement: Supplementary file 1 [file ijms-26-00644-s001.zip › Figures TEM without color musk/Figure 7/7B.tiff]

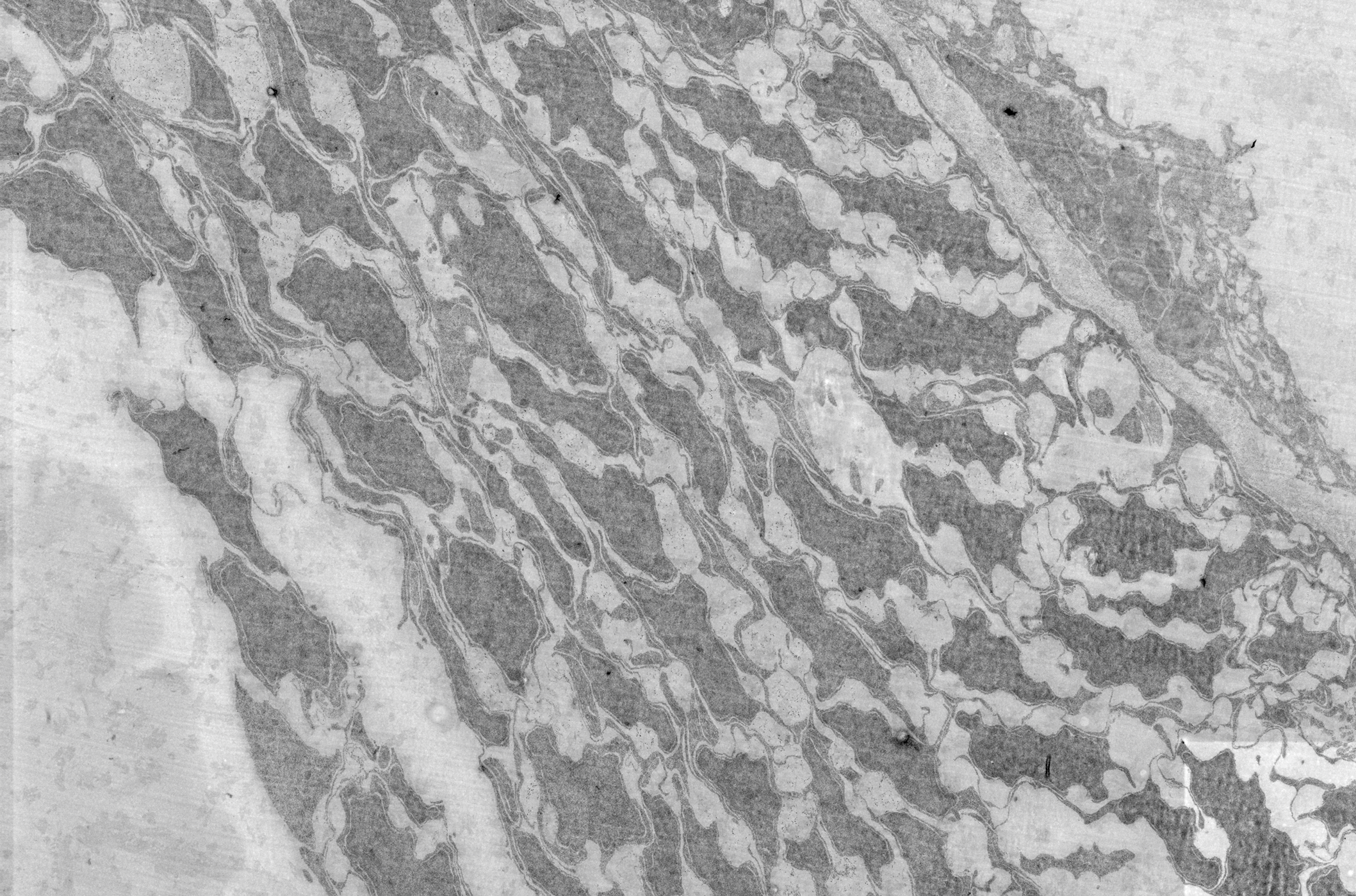

Supplement: Supplementary file 1 [file ijms-26-00644-s001.zip › Figures TEM without color musk/Figure 7/7A.tiff]

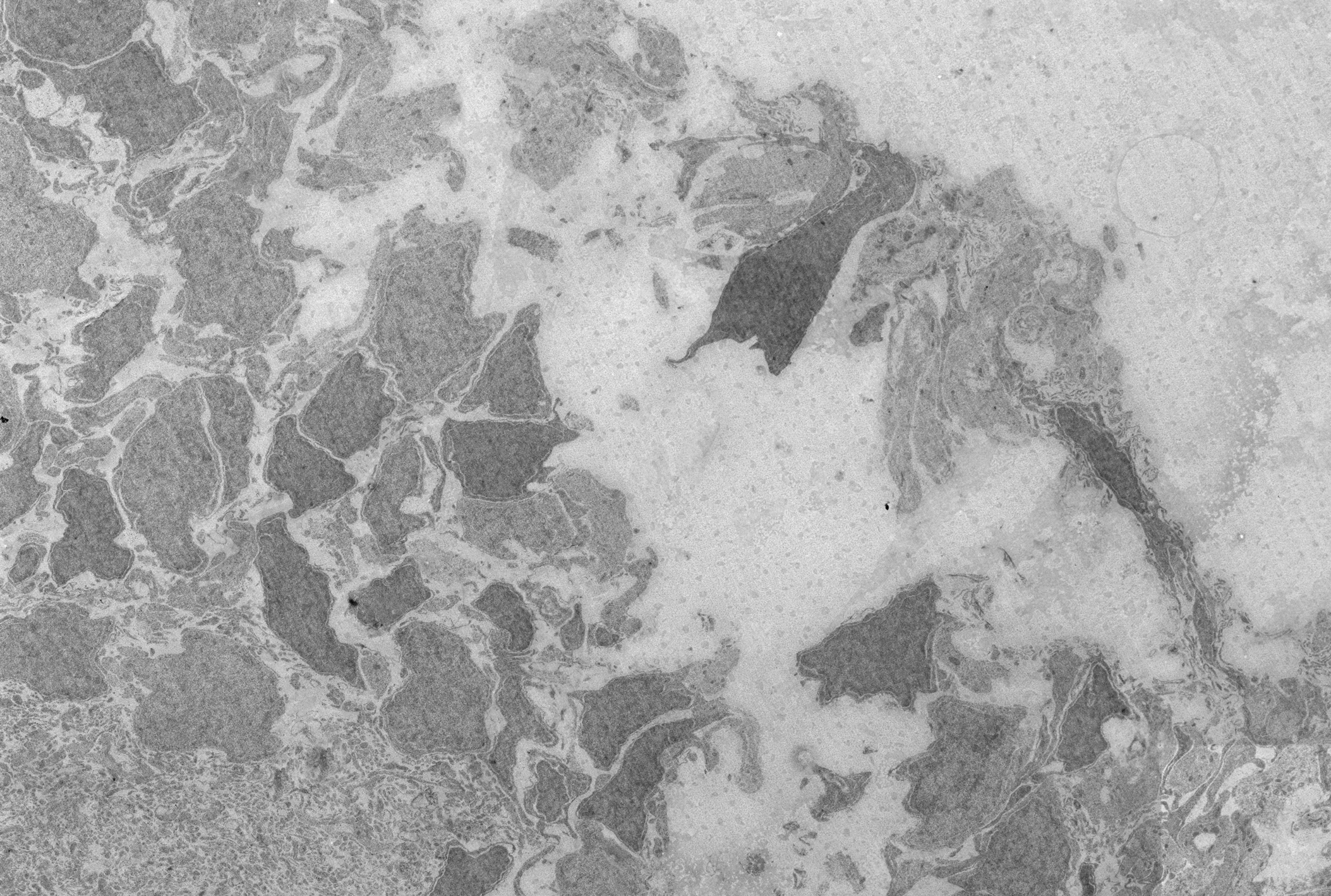

Supplement: Supplementary file 1 [file ijms-26-00644-s001.zip › Figures TEM without color musk/Figure 7/7C.jpg]

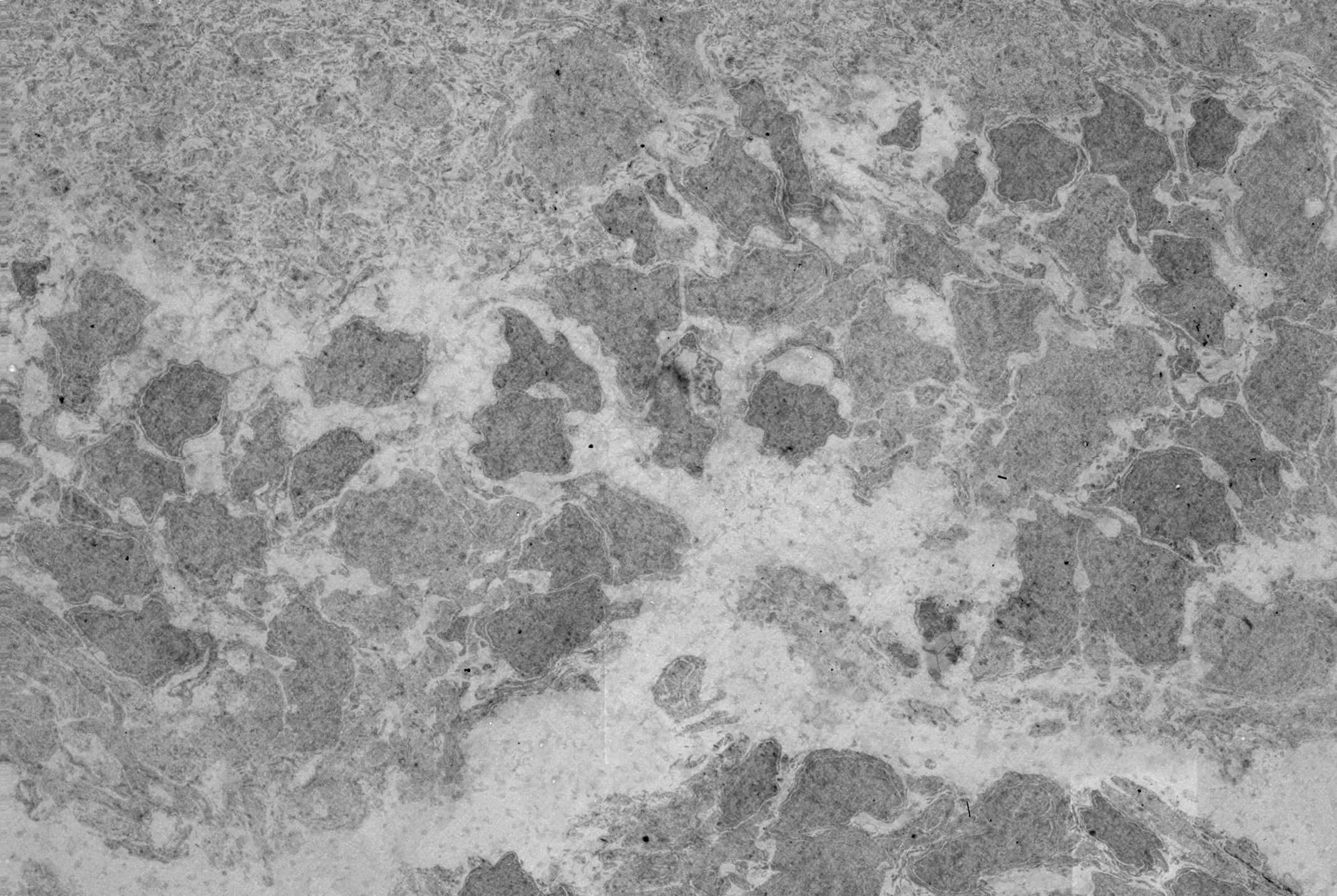

Supplement: Supplementary file 1 [file ijms-26-00644-s001.zip › Figures TEM without color musk/Figure 9/9A.tiff]

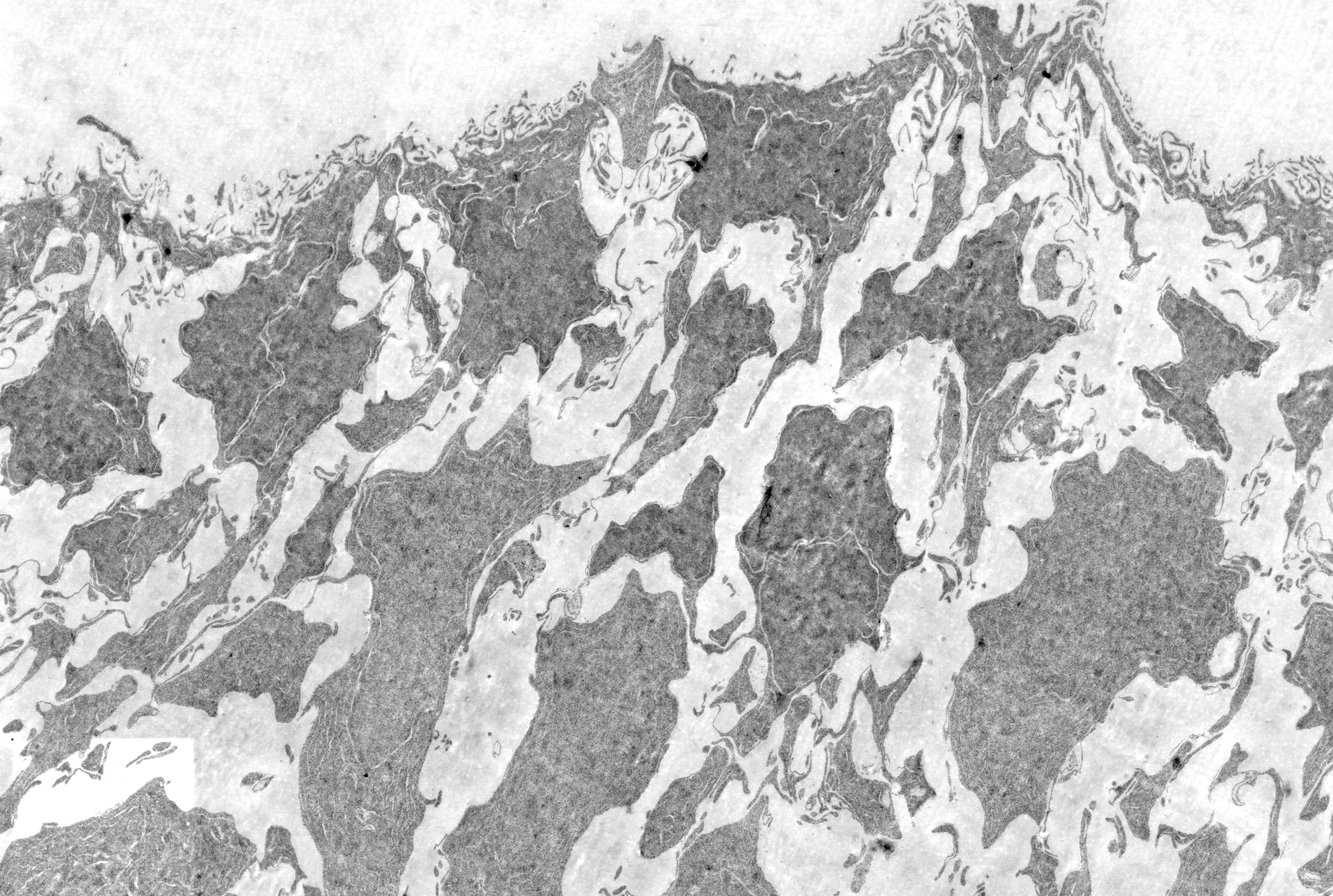

Supplement: Supplementary file 1 [file ijms-26-00644-s001.zip › Figures TEM without color musk/Figure 9/9F.tiff]
